# Synergistic Effect of Blended Precursors and Silica Fume on Strength and High Temperature Resistance of Geopolymer

**DOI:** 10.3390/ma17122975

**Published:** 2024-06-18

**Authors:** Bosong Cao, Yi Li, Peipeng Li

**Affiliations:** 1 School of Civil Engineering and Architecture, Wuhan University of Technology, Wuhan 430070, China; 2 Sanya Science and Education Innovation Park, Wuhan University of Technology, Sanya 572019, China

**Keywords:** geopolymer, high temperature resistance, blended precursor, silica fume

## Abstract

This paper investigates the high temperature resistance performance and mechanism of potassium-activated blended precursor geopolymer with silica fume. The failure morphology, volume, and mass loss, compressive strength deterioration, hydration production, and pore structure are measured and analyzed. The results show that introducing slag into fly ash-based geopolymer could greatly improve the 28 d compressive strength but reduce the thermal stability. In contrast, the partial substitution of fly ash by metakaolin contributes to excellent high temperature resistance with slightly enhanced 28 d compressive strength. After being exposed at 800 °C, the residual compressive strength of F7M3 remains at 37 MPa, almost 114% of the initial ambient-temperature strength. An appropriately enlarged silica fume content in geopolymer results in increased compressive strength and enhanced thermal stability. However, an excessive silica fume content is detrimental to the generation of alkali-aluminosilicate gels and ceramic-like phases and thus exacerbates the high temperature damage.

## 1. Introduction

The construction sector has witnessed an increased global recognition of the need for environmental preservation in recent years, leading to a greater emphasis on sustainability and carbon reduction. In response, researchers have been actively investigating environmentally friendly and low-carbon building materials as a means to counterbalance substantial energy consumption and carbon emissions [1,2]. A significant advancement in this field occurred in 1978 when Davidovits proposed the concept of the “geopolymer”, a novel type of cementitious material. It is derived from precursor materials such as metakaolin, fly ash, and slag, which undergo a process known as “dissolution-monomer reconstruction-polycondensation” in the presence of alkaline activators [3,4]. Eventually, a three-dimensional network structure in geopolymers is formed by [SiO_4_] tetrahedra and [AlO_4_] tetrahedra [5]. Ongoing investigations have revealed that geopolymers possess numerous commendable characteristics, including rapid hardening, early strength development, high compressive strength, and remarkable high temperature resistance [6,7,8,9,10]. In the contemporary era, the geopolymer has garnered considerable attention as a highly promising cementitious material with the capacity to substantially decrease CO_2_ emissions during its manufacture [11]. Furthermore, when compared to conventional Portland cement, geopolymers exhibit improved fire resistance, indicating a potential paradigm shift in the requirements for building materials [12,13,14].

The current dominant trend in geopolymer research revolves around the utilization of a single precursor material [15]. The geopolymers derived from fly ash particularly demonstrate superior thermal stability at elevated temperatures. However, utilizing a single precursor material often presents challenges in achieving a balance between mechanical strength and high temperature resistance. The geopolymer produced from fly ash, slag, and metakaolin exhibits distinct advantages, namely less thermal degradation, high strength, and an efficient viscous sintering capacity under elevated temperatures, respectively. However, it is important to note that these materials also possess certain drawbacks, such as curing difficulty and reduced strength, significant high temperature decomposition, and extreme pore pressure under high temperatures, respectively [16,17,18]. Although the heating curing could accelerate the reaction kinetics and bolster strength, it complicates the preparation process and hinders its practical implementation in engineering projects. The geopolymerization process can be optimized and the strength of geopolymer pastes can be enhanced by adding slag and metakaolin in fly ash to form a blended precursor [19,20,21]. Many studies currently emphasize the substitution of fly ash with different proportions of metakaolin and slag to investigate the optimal formulation, while other parameters remain fixed. However, this approach ignores the interactions between the precursor, the hydrogel ratio, and the activator. It may be difficult to obtain a formulation with the best overall performance. Currently, there is a lack of holistic studies on formulation design, considering the physicochemical properties of the precursors, the optimum water-to-binder ratio, and the alkali equivalent. A holistic approach considering the physicochemical traits of the precursors, optimum water-binder ratio and alkali equivalents may enhance both precursor utilization and the high temperature resistance of geopolymers.

Besides precursor materials, some researchers have studied the potential use of various by-products in Supplementary Cementitious Materials (SCMs) as reactive additives for improving geopolymers’ mechanical and thermal resistance [22,23]. Silica fume (SF) is a by-product of the silicon smelting process. Due to the excellent pozzolanic activity, silica fume (SF) is widely used for enhancing the mechanical properties and durability of concrete materials [24,25]. F.N. Okoye et al. pointed out that silica fume enhances the compressive, tensile, and flexural strength of geopolymers by increasing the densification of the matrix [26]. Chindaprasirt et al. found that the presence of reactive silica fume contributed to increasing the reactivity of the system, thereby improving geopolymers’ resistance to magnesium sulfate and sulfuric acid attacks [27]. In addition, studies have shown that the incorporation of silica fume alleviates the high temperature damage and improves the thermal resistance of geopolymers, by optimizing the microstructure [28]. Yang et al. indicated that silica fume also contributes to the enhancement of the bulk stability and residual strength of geopolymers at high temperatures by affecting the thermal shrinkage, densification, and potential cracking [29].

The effect of the single precursor- and silica fume-based geopolymer composites has been reported in previous studies. However, the synergistic effect of silica fume and blended precursors on the mechanical and high temperature resistance properties of geopolymers has not been adequately studied. Thus, it is imperative to comprehend the synergistic effect of silica fume on the high temperature resistance of blended precursor geopolymers to optimize their mix design and application. In this paper, the blended precursor system was formed by partially replacing fly ash with either metakaolin or slag. The geopolymer was subsequently synthesized by activating the designed blended precursor with an optimized water-to-binder ratio and potassium activator at ambient temperature. Then, the effects of silica fume on the high temperature resistance and mechanism of the blended precursor geopolymer were investigated and analyzed. It could provide theoretical bases for designing sustainable fire-resistant building materials and promoting the application of geopolymers in practical engineering.

## 2. Experimental Program

### 2.1. Raw Materials

**Figure 1:**
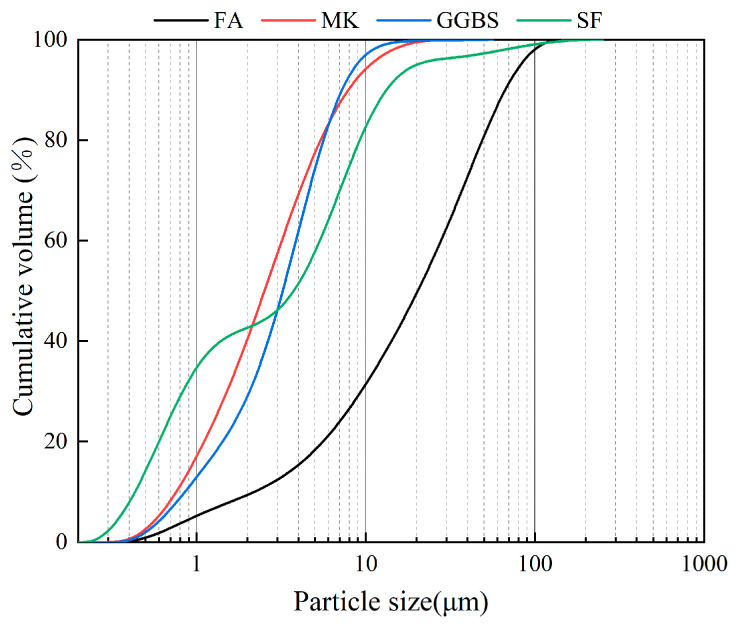
The particle size distributions of raw materials.

The precursor materials utilized in this study were F-grade fly ash (FA), ground granulated blast slag (GGBS), metakaolin (MK), and silica fume (SF). Fly ash and ground granulated blast slag were produced in Henan, China; metakaolin and silica fumes were made in Wuhan, China. The alkali activator consisted of potassium hydroxide (99% KOH) and potassium silicate (water glass, 26.88% SiO_2_, and 12.75% K_2_O). Potassium hydroxide and potassium silicate were both produced by China National Pharmaceutical Group. The main raw materials and their chemical compositions are exhibited in Table 1. The particle size distributions of the precursor materials obtained by the laser particle size analyzer are shown in Figure 1.

### 2.2. Mix Design and Preparation

Table 2 shows the mix design of the geopolymers. The alkali activator was synthesized through the combination of potassium hydroxide granules, potassium silicate solution, and distilled water. All dry solid powders were first mixed to a homogenous state using a concrete mixer, and then the alkali-activated solution was gradually added. After stirring at low speed for 30 s, the mixture was stirred at high speed for 120 s. The specimens were cast in molds (40 mm × 40 mm × 40 mm) after 24 h in a sealed condition, and were then demolded and cured in standard conditions (temperature 20 ± 2 °C, relative humidity ≥ 95%) for 28 d.

The flowability of geopolymer is an important index that affects its application in real engineering in many aspects, such as pumping performance, self-compaction properties, and set hardening. The flowability of a geopolymer is usually reduced by the addition of metakaolin, slag, and silica fume, which have high water absorption capacities [30]. In addition, it should be noted that water has a crucial role in determining the high temperature mechanical properties and strength of geopolymers [31]. In this study, we first determined the optimal water-to-binder ratios for each of the single precursor (FA, MK, GGBS) geopolymers, and then calculated new water-to-binder ratios based on the proportion of different precursors in the samples. The flowability of geopolymer specimens was controlled to be within similar ranges, as can be seen in Table 2, to satisfy the essential requirements in practical engineering. The alkaline modulus (Ms, SiO_2_/Na_2_O molecular ratio) of the activator was set constant as 1.0 for all samples, and the alkali equivalent K_2_O% chosen for the experiments was previously determined based on the proportions of the different precursor materials. Thus, sufficient polymerization, improved workability, and improved high temperature resistance can be achieved. The substitution ratios of FA by GGBS or MK were set to 10%, 30%, and 50%. The water in the samples consisted of distilled water and water in the base activator. 

### 2.3. Testing Methods

#### 2.3.1. Flowability Test

The spread flow of fresh geopolymer samples were measured by a mini cone (top diameter 70 mm, bottom diameter 100 mm, height 60 mm) fluidity test. The cone was fully filled with fresh geopolymer, then slowly vertically lifted at a constant speed. The fresh geopolymer freely flowed for around 30 s, and the average flow diameters from two perpendicular directions were recorded.

#### 2.3.2. Volume and Mass Loss Test

The volume and mass of geopolymer specimens were measured before and after exposure to elevated temperatures by a vernier caliper and an electronic scale, respectively. Then, the volume shrinkage and residual mass ratio of the geopolymer specimens were calculated.

#### 2.3.3. Compressive Strength Test

To reveal the degradation profile of the mechanical properties of geopolymer after high temperature, the compressive strength of samples before and after exposure to high temperature was measured using a universal testing machine (TSE300kN, WANCE, Shenzhen, China). Three specimens for each group were tested to achieve accurate results.

#### 2.3.4. High Temperature Test

The cured geopolymer specimens at 28 d were respectively heated to 200 °C, 400 °C, 600 °C, 800 °C using a muffle furnace at a heating rate of 10 °C/min. After reaching the target temperature, the specimens were subjected to the desired temperature for another 1 h. Then, the heating process was terminated, and the furnace and samples were finally cooled to room temperature through the natural cooling process.

#### 2.3.5. X-ray Diffraction Test

The geopolymer samples were ground to powder state, and the X-ray diffraction analysis of powders was carried out before and after exposure to high temperature with Panalytical Empyrean to determine mineralogical phases. The system was operated in a continuous scan mode in the range 2*θ* of 10°–70° with a scan speed of 5°/min.

#### 2.3.6. Thermogravimetric Test

A thermogravimetric analysis (TGA) of the geopolymer was carried out with PerkinElmer STA 6000 to assess its thermal stability. The geopolymer specimens on platinum discs were heated from 30 °C to 800 °C with a heating rate of 10 °C/min under a nitrogen atmosphere.

#### 2.3.7. Mercury Intrusion Porosimetry Test

To observe the pore structure of geopolymer specimens, the mercury intrusion porosimetry (MIP) analysis was performed using a mercury porosimeter (AutoPore IV 9520, Micromeritics). The geopolymer specimens were crushed into small pieces ranging in size from 2 to 4 mm for the MIP test.

#### 2.3.8. Scanning Electron Microscopy Test

The scanning electron microscopy (SEM, TESCAN MIRA LMS, Czech) test was used to observe the microscopic morphology of the geopolymer specimens before and after exposure to high temperatures. Before the SEM analysis, the test samples were first immersed in isopropanol for 24 h and subsequently dried at 60 °C for 24 h. The dried sample was then coated with Au using a sputter coater with a current of 40 mA for 30 s.

## 3. Results and Discussion

### 3.1. Macroscopic Morphology and Failure Mode

**Figure 2:**
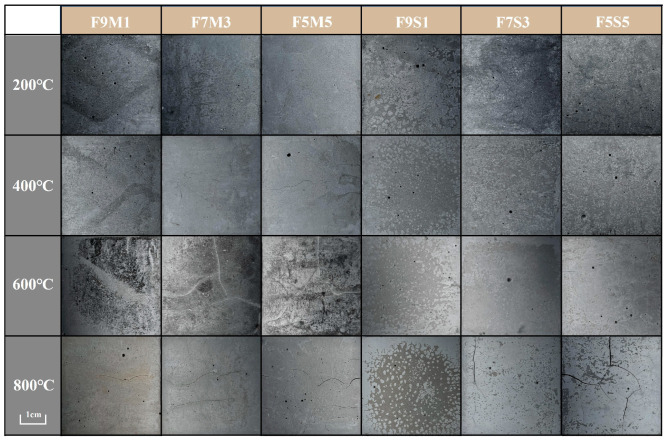
Macroscopic morphologies of geopolymers after high temperatures.

**Figure 3:**
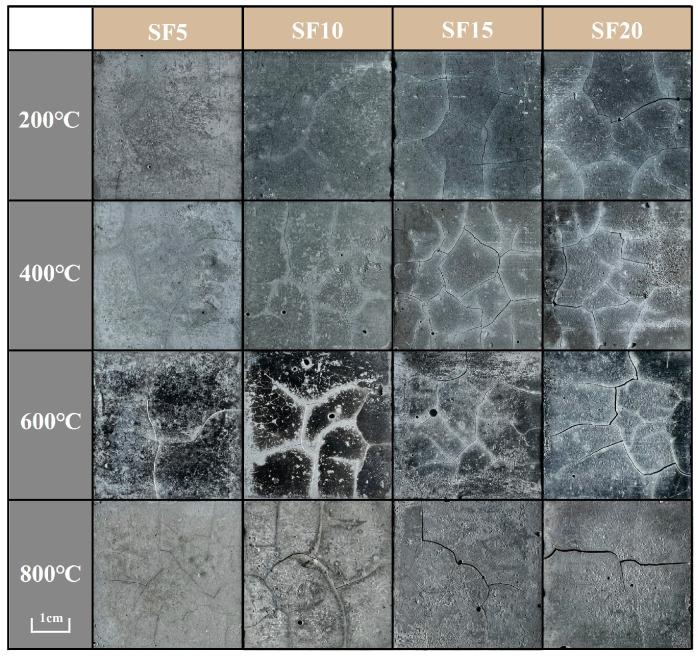
Macroscopic morphologies of geopolymers with SF.

Figure 2 and Figure 3 illustrate the changes in the macroscopic morphologies of the designed blended precursor geopolymers exposed to various elevated temperatures. The presence of pores on the surface of the geopolymers is attributed to the water evaporation that occurred within the temperature range of 200 °C to 400 °C. The phenomenon shown in Figure 2 shows is significant in fly ash–slag-based geopolymers. Visible cracks occur in the fly ash–metakaolin-based geopolymer at 600 °C. When subjected to a temperature of 800 °C, the geopolymers with metakaolin exhibited grey surfaces without alteration in crack patterns. The ash–slag geopolymer started to develop cracks after exposure to 800 °C, suggesting it has a poorer thermal stability than the fly ash–metakaolin geopolymers.

The water vapor cannot effectively be released from the dense matrix in a short time, resulting in a sharply increased pressure in the microcracks and eventually rupture [32]. The introduction of SF in the fly ash–metakaolin geopolymers leads to the development of cracks, and with increasing SF content, the number of cracks increases (Figure 3). The observed effect can be attributed to the increased water adsorption capacity of silica fume, which results in a greater amount of free water in the matrix. Consequently, the detrimental impact of water evaporation on the microstructure is intensified.

**Figure 4:**
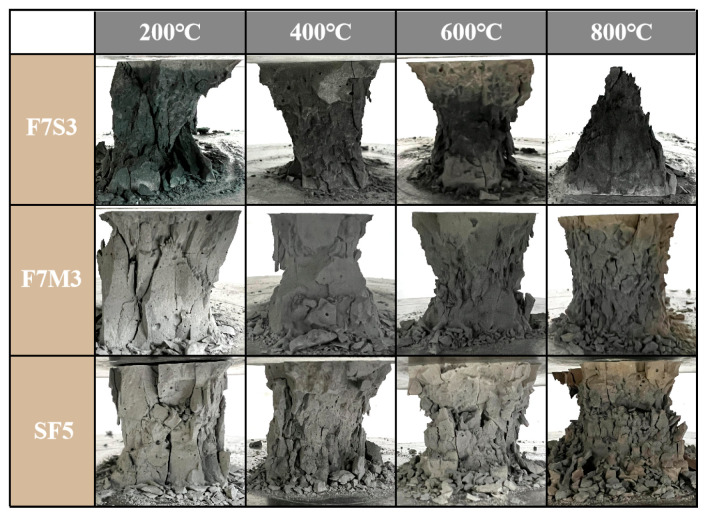
Compressive failure modes of geopolymers after high temperatures.

The three types of samples with the best residual mechanical properties were selected to analyze the influences of different precursors and silica fume content on the compressive failure modes. Figure 4 shows the compressive failure modes of selected specimens after calcination at various temperatures. The collapse damage of the F7S3 is more obvious and severe, accompanied by other debris during its destruction. While the F7M3 and SF5 maintain relatively complete damage patterns. 

### 3.2. Volume Shrinkage and Mass Loss

**Figure 5:**
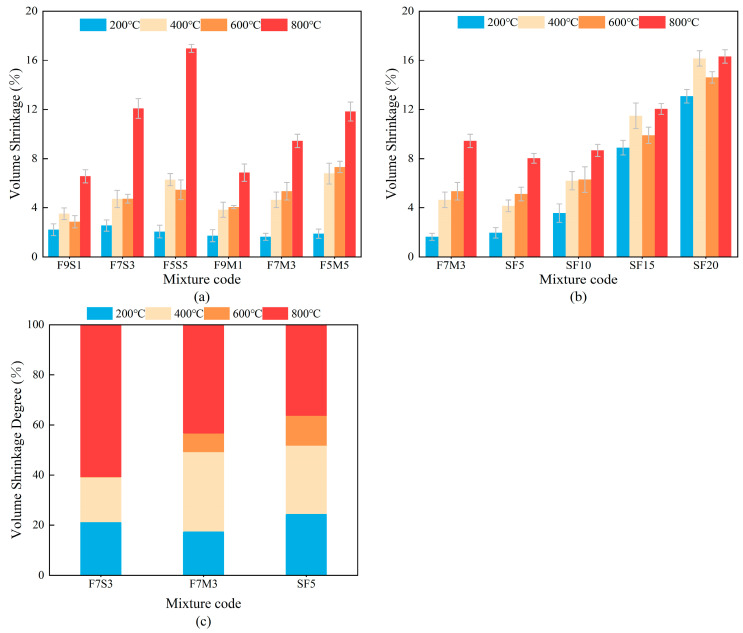
Volume shrinkages of geopolymers exposed to high temperatures (**a**) different precursors, (**b**) different silica fume contents, (**c**) volume shrinkage degree.

Figure 5 shows the volume shrinkage of the designed blended precursor geopolymers after undergoing calcination at different temperatures. The fly ash–slag-based geopolymers have higher water-to-binder ratios and show higher volume shrinkages compared to the fly ash–slag-based specimens, which can be attributed to the stable gel structure and low calcium content [30]. Figure 5 shows that the volume shrinkage of SF5 is overall smaller than that of F7M3 at high temperatures, which is due to the enhancement of volumetric thermal stability of the ground polymer by the silica fume [28]. The volume shrinkage of the geopolymer with silica fume in Figure 5b increases with the increase in silica fume content, which is especially noticeable at 200 °C. Incorporating silica fume increases the free water content and causes a greater degree of water evaporation, ultimately leading to increased volume shrinkage at 200 °C. The reduction in volume shrinkage in SF15 and SF20 is due to the high temperature expansion of partially unreacted reactive silica [33]. 

Figure 5c shows the development process of volume shrinkage in the geopolymer at high temperatures. The volume shrinkage of the samples should be from 400 °C to 600 °C, as most of the free and bound water has been evaporated. While It is noteworthy that the volume shrinkages of some geopolymers initially rise and thereafter decline from 200 °C to 600 °C. On the one hand, the high temperature-induced water evaporation and thermal cracking result in many pores and microcracks that generate unstable gel structures. On the other hand, the hot smoke invading the cracks and pores rapidly increases the temperature of the gel in their vicinity, which promotes crack development within the sample as a whole. The decrease in the volume shrinkage does not represent the true shrinkage of the sample. There were samples that cracked at high temperatures and the measurements included the space between the cracks as well as the core of the sample.

This hypothesis might be supported by the crack development observed in Figure 2 and Figure 3. The shrinkage generated at 800 °C accounts for the largest portion of the total shrinkage, which can be attributed to high temperature viscous sintering.

**Figure 6:**
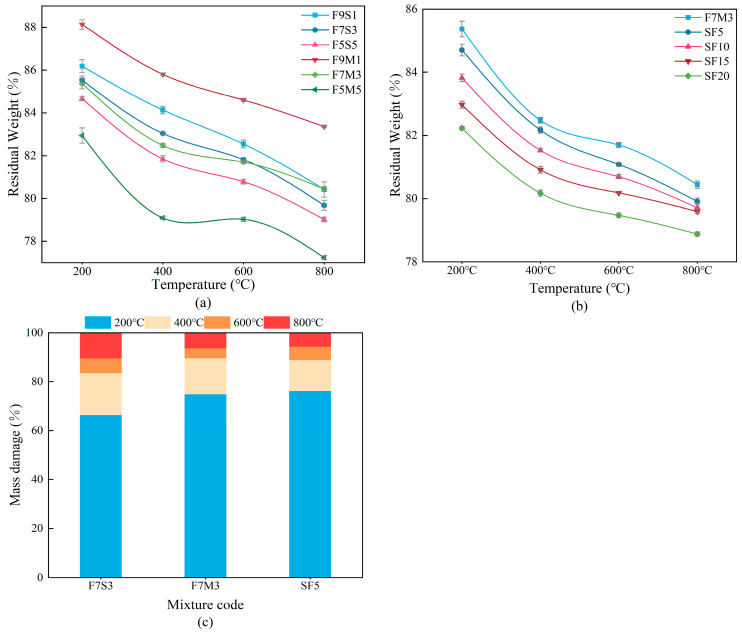
Mass losses of geopolymers after calcination (**a**) different precursors, (**b**) different silica fume contents, (**c**) degree of mass loss.

Figure 6 shows the mass loss of the designed geopolymers after high temperature exposure. The mass loss rate in Figure 6a increases with decreasing fly ash incorporation, which is in line with the literature [30]. Figure 6b shows that the incorporation of silica fume mainly exacerbates the mass loss at around 200 °C. The differences in the macroscopic mass loss are attributed to the varying free water contents and evaporations that occurred at around 100 °C. Figure 6c shows that the mass loss from the geopolymers after 400 °C exposure accounts for over 80% of the total mass reduction, due to the evaporation of both free and chemically bound water. The free and bound water generally evaporate at temperatures of 100 °C–300 °C and 300 °C–600 °C, respectively, because more heat must be absorbed by the bound water during evaporation [34].

### 3.3. Compressive Strength

**Figure 7:**
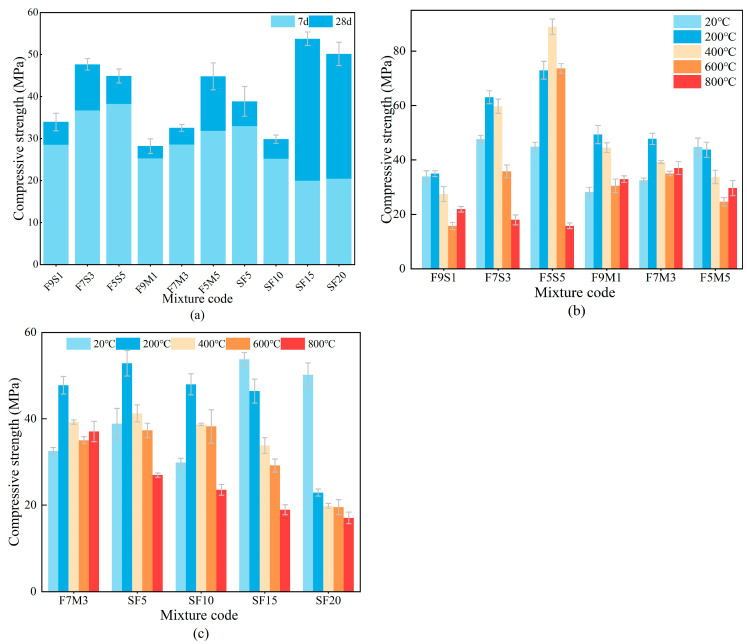
Compressive strength of geopolymers at different temperatures (**a**) curing ages, (**b**) different precursors, (**c**) different silica fume contents.

The compressive strength of geopolymers cured for 7 d and 28 d without calcination is presented in Figure 7a. The partial substitution of fly ash by metakaolin or slag could enhance the compressive strength of geopolymers, which can be attributed to the high calcium contents that lead to the extensive formation of C-S-H gels and dense microstructures [16]. Furthermore, the metakaolin contributes to offering more [AlO_4_] tetrahedra, facilitating the geopolymerization process and the formation of aluminosilicate gel [20]. As the silica fume content in Figure 7a increases, the compressive strength of the geopolymer initially increases, subsequently decreases, and then rises again. The observed phenomenon can be attributed to the combined physical and chemical characteristics of silica fume. The particle size of silica fume is very small, and it can fill the pores through the stacking effect and enhance the densification and mechanical properties of the geopolymer. In addition, the activated silica in the silica fume can sufficiently participate in the geopolymerization process, under the action of the alkali activator. Silica fume provides active silicon and has a positive effect on the geopolymerization process and compressive strength [29,33]. However, when the silica fume content surpasses a certain threshold, the silica fume consumes a substantial amount of OH^−^, leading to insufficient dissolution of the precursor [33] and reduced compressive strength of the geopolymer [35,36]. Simultaneously, the silica fume absorbs a considerable amount of free water and makes the geopolymer more viscous. Thus, it hinders the ions’ transport and declines the geopolymerization process, and it also results in enlarged shrinkage, deformation, microcracks, and early-stage damage [30,37]. As the silica fume content continues to rise further, there is a slight enhancement in compressive strength, probably due to the fine filler effect to refine the pore structure [38,39].

Figure 7b shows the compressive strength results of the blended precursor geopolymers from F9S1 to F5M5 after calcination temperatures between 20 °C and 800 °C. The compressive strength of geopolymers, especially those incorporated with slag, experiences an improvement at 200 °C, mainly attributed to the further hydration production generation from remaining unreacted precursors under high-temperature conditions. However, a higher calcination temperature (e.g., 600 °C) would harm the compressive strength because of the partial gel dehydroxylation [40]. In the case of even higher temperatures, e.g., 800 °C, the dissolution of C-(A)-S-H gels and the negative effect of expansive crystalline phases such as akermanite greatly exacerbate compressive strength loss [17]. The fly ash–metakaolin-based geopolymers exhibit much a higher residual compressive strength after exposure temperature of 800 °C, indicating superior high-temperature resistance. Because metakaolin has abundant Al, it facilitates the generation of ceramic-like phases and partly compensates for strength loss. 

Figure 7c presents the compressive strength of the geopolymers with different silica fume content exposed to temperatures ranging from 20 °C to 800 °C. The residual compressive strengths of the geopolymers show an increasing and then decreasing trend with the increase in silica fume content before 800 °C. In particular, a decreasing trend can be observed in the residual compressive strength of the geopolymer with silica fume at 800 °C. The observed phenomenon is probably due to the fact that silica fume enhances the compactness of the geopolymer, which exacerbates the damage to the matrix due to water evaporation. Meanwhile, a too-high silica fume content would raise the temperature of gel crystallization and delay gel formation [33,37]. Moreover, the presence of microcracks induced by unhydrated binder particles probably exacerbates the high temperature damage.

### 3.4. X-ray Diffraction Analysis

**Figure 8:**
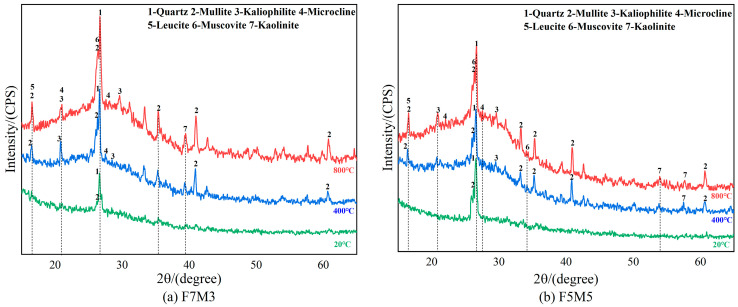

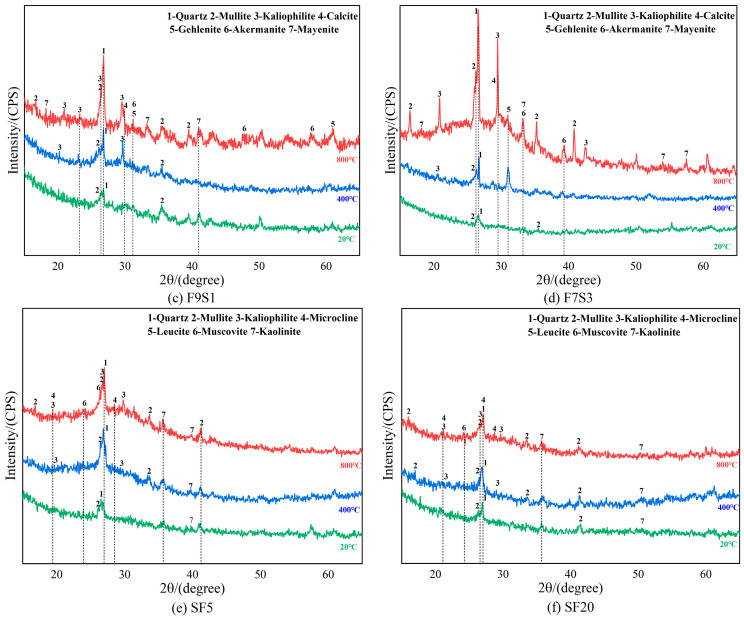
XRD patterns of geopolymers exposed at different temperatures.

The XRD patterns of the designed geopolymers after different temperatures are presented in Figure 8. Before exposure to high temperatures, the main crystalline phases of the geopolymer are quartz and mullite. The intensity of the diffraction peaks in the crystalline phases of geopolymers such as kaliophilite and mullite in F7M3 and F5M5 is significantly increased at 800 °C, and the leucite phase is also detected, as illustrated in Figure 8a,b. These high temperature-resistant ceramic-like phases (kaliophilite, microcline, leucite) can enhance thermal stability and residual strength after high temperatures [30,33]. The intensity of most diffraction peaks from F7M3 is also higher than that from F5M5, which is linked to the enlarged generation temperature of the crystalline phase by the metakaolin [29,41]. The typical C-S-H gels are not seen in the XRD patterns of the fly ash–slag-based geopolymers, as shown in Figure 8c,d, because the Ca^2+^ ion content is insufficient to form a C-S-H gel in the case of high concentration of the alkali and a low slag content. Instead, it is substituted for the alkali metal cation (M) in the geopolymers to form the hybrid M-(C)-A-S-H gel [42]. At 800 °C, the productions of akermanite and gehlenite in fly ash–slag-based geopolymers lead to a more porous microstructure and severe strength loss at high temperatures [43,44].

The reduction in the peak intensity of the crystalline phases is observed with the increase in the silica fume content, as depicted in Figure 8e,f. Some findings indicate that increasing the silica fume content can improve the strength of geopolymers at high temperatures [28]. Compared to the pure fly ash-based geopolymer, the blended precursor geopolymer prepared from fly ash and metakaolin has a higher requirement for both essential activators and water. Consequently, the consumption of excessive OH^−^ by silica fume could aggravate insufficient geopolymerization process and then prevent the generation of crystalline phases at high temperatures [45]. Furthermore, the insufficient geopolymerization process also demonstrates that the higher compressive strength of the geopolymers with silica fume is mainly attributed to the physical filling effect of SF on the matrix.

### 3.5. Thermal Analysis

**Figure 9:**
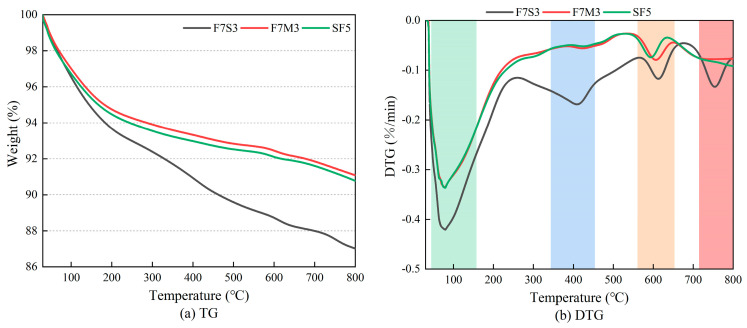
TG-DTG analysis of geopolymers (**a**) TG, (**b**) DTG.

Figure 9 presents the TG and DTG curves of F7S3, F7M3, and SF5 after 28 days, which reflect the crystalline phase transitions of the geopolymers at different temperatures. The weight reduction obtained from the TG-DTG test is in accordance with the macroscopic sample mass loss discussed in Section 3.2. Because there is no holding time at the target temperatures in the TG-DTG test, the degree of weight reduction in Figure 9a is relatively lower than the mass loss. Figure 9b presents several distinct weight loss peaks. The weight loss peaks consistently emerge around 100 °C, due to the free water evaporation and the initial dehydration of hydrated aluminosilicate. The broad nature of the former peak might overshadow the endothermic peak of the latter, leading to a merged peak pattern evident in most samples. Notably, two extra dominant peaks in F7S3 can be observed, around 400 °C primarily corresponding to Ca(OH)_2_ dehydration and 750 °C linked to calcite decomposition. The weight loss peak observed around 600 °C in all samples likely arises from the dehydroxylation or crystallization of the gel.

### 3.6. SEM Analysis 

**Figure 10:**
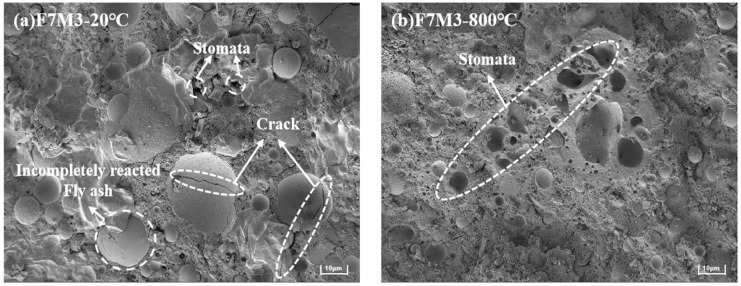

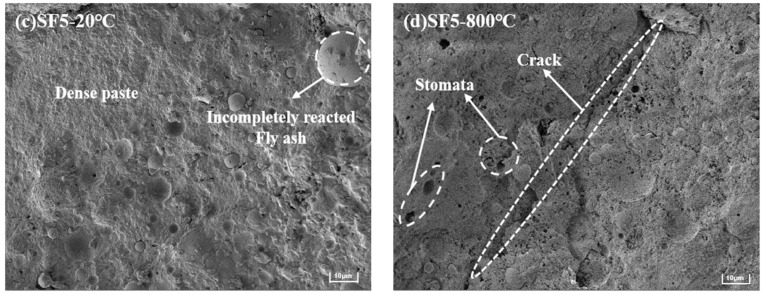
SEM morphologies of geopolymers (**a**) F7M3-20 °C, (**b**) F7M3-800 °C, (**c**) SF5-20 °C, (**d**) SF5-800 °C.

In Figure 10, the SEM analysis shows the microstructure morphologies of geopolymers before and after exposure to high temperatures. Compared with sample SF5, the microstructure of F7M3 is looser at room temperature. A few pores and unreacted precursor particles are still distributed outside the amorphous alkali aluminosilicate gel. While SF5 in Figure 10c has a dense microstructure and a significant size reduction of the unreacted fly ash particles. The reactive SiO_2_ provided by silica fume can enhance the reactivity of the alkali-activated system, which promotes precursor dissolution and contributes to rich alkali aluminosilicate gels [37]. 

Figure 10b shows large pores and incompletely reacted fly ash in F7M3 after exposure to 800 °C. Furthermore, thermal cracking at high temperatures leads to pore collapse and macropore connectivity. However, there is evidence that partial pores have been filled and healed, as shown by dense features. The geopolymer would then undergo a melting process, resulting in the formation of a compact ceramic structure that fills the internal microcracks in the calcination process above 700 °C [20,46].

Figure 10d shows the microstructure of SF5 after exposure to 800 °C, characterized by a denser matrix with few pores compared to F7M3 at 800 °C. It indicates that an appropriately high silica fume content could reduce geopolymer porosity and mitigate pore collapse at high temperatures. While there are more microcracks or even penetrating cracks distributed throughout the matrix in SF5. Excessive silica fume content retards gel generation and increases the crystallization temperature of gel, which hinders the viscous sintering at 800 °C and weakens the microcrack healing ability.

### 3.7. Pore Structure Analysis

**Figure 11:**
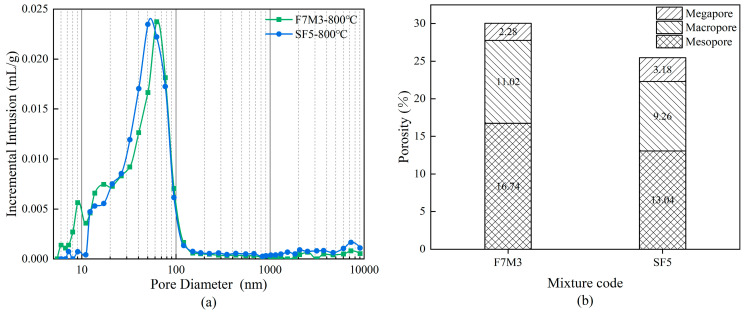
Pore size distributions of geopolymers, (**a**) pore size distribution, (**b**) porosity.

Figure 11 shows the pore size distribution in F7M3 and SF5 calcinated at 800 °C observed by MIP. The pore size distribution of F7M3 calcinated at 800 °C shows a predominant range of pore size below 100 nm with a critical diameter of around 60 nm. The SF5 has a similar pore size distribution range but a slightly smaller critical diameter of 50 nm. Compared with F7M3, the pore size of SF5 in Figure 11a shows a decreasing tendency, which is attributed to the reduction of pore size by silica fume through the promotion of gel generation and physical filling effect. Generally, the critical pore size of geopolymers will increase with increasing temperature, which is due to evaporation of bound water, microcrack development and gel decomposition [16,33]. In addition, mesopore collapse due to crack development and capillary pressure can also promote large pores propagation [47]. 

To identify the high temperature induced pore structure change on different scales, the pore fractions are further divided into three categories: mesopore (2–50 nm), macropore (50–7500 nm), and megapore (>7500 nm) [48]. Figure 11b illustrates the porosity and pore distribution of the geopolymers after exposure to high temperatures. The SF5 has a lower porosity compared to F7M3. However, the compressive strength of SF5 at 800 °C is 25 MPa, which is lower than the 37 MPa of F7M3. Moreover, the XRD pattern of SF5 showed a significant decrease in the diffraction intensity of the ceramic-like crystalline phase, compared to F7M3. Combined with the residual compressive strength after high temperatures and chemical composition analysis, the viscous sintering of SF5 at high temperatures is insufficient. Therefore, the smaller volume of mesopores in SF5 is primarily due to the filling of pores at room temperature. The porosity occupied by the macropores in SF5 is also lower than that of F7M3, namely 9.26%, indicating that the stronger thermal stability of SF5 reduces the pore collapse at high temperatures [38]. 

## 4. Conclusions

This paper aimed to develop the blended precursor geopolymers by partial substitution of fly ash by ground granulated blast furnace slag or metakaolin and the mechanisms of silica fume action on the high temperature resistance of blended precursor geopolymers are investigated. The following conclusions can be drawn:

- The development of blended precursor geopolymers by partially substituting fly ash with slag significantly benefits dense structures, 28 d compressive strength, and residual strength below moderate temperature exposure. However, the creation of the M-(C)-A-S-H hybrid gel due to higher calcium contents adversely reduces the thermal stability leading to enlarged volume shrinkage and mass loss at high temperatures. The residual compressive strength dramatically declines after being exposed to high temperatures such as 800 °C due to the generation of expansive akermanite and gehlenite to result in porous microstructure.
- The blended precursor geopolymers prepared from fly ash and metakaolin have favorable mechanical properties and excellent high-temperature resistance. The large amount of reactive Al_2_O_3_ in metakaolin promotes the formation of silica-aluminate gels and enhances the microstructure and compressive strength of the geopolymers. Large gels generate kaliophilite by viscous sintering to compensate for the strength loss at high temperatures.
- An appropriate silica fume content in the blended precursor geopolymer contributed to reduced porosity and enhanced compressive strength and mitigates pore collapse at high temperatures. However, excessive silica fume content reduced the residual compressive strength of the blended precursor geopolymers at high temperatures due to the generation of alkali aluminosilicate gels and ceramic-like phases being hindered. In addition, the presence of more free water increases the mass loss and volume shrinkage of geopolymers which include silica fume.

## Figures and Tables

**Table 1:** Chemical composition of FA, MK, GGBS, and SF.

Oxide (%)	SiO_2_	Al_2_O_3_	CaO	MgO	Fe_2_O_3_	K_2_O	TiO_2_	Others	LOI
FA	60.91	31.09	3.29	0.38	0.3	1.29	0.92	1.82	4.66
MK	52.09	46.03	0.10	0.12	0.26	0.11	0.95	0.34	1.10
GGBS	30.16	19.60	35.32	9.31	1.99	0.35	0.48	2.79	4.84
SF	97.51	0.16	0.38	0.88	-	0.29	-	0.78	3.59

**Table 2:** Mix proportions and flowability of the geopolymer samples.

Sample Code	FA (wt.%)	MK (wt.%)	Slag (wt.%)	SF (wt.%)	K_2_O% ^a^ (wt.%)	w/b	Si/Al Ratio	Flowability (mm)
F9M1	90%	10%	0%	0%	9.2%	0.18	3.44	235
F7M3	70%	30%	0%	0%	11.6%	0.27	3.14	225
F5M5	50%	50%	0%	0%	14.0%	0.34	2.88	215
F9S1	90%	0%	10%	0%	7.7%	0.205	3.56	225
F7S3	70%	0%	30%	0%	7.1%	0.23	3.46	227
F5S5	50%	0%	50%	0%	6.5%	0.245	3.33	232
F7M3_SF5_	66.5%	28.5%	0%	5%	11.6%	0.27	3.41	-
F7M3_SF10_	63%	27%	0%	10%	11.6%	0.27	3.71	-
F7M3_SF15_	59.5%	25.5%	0%	15%	11.6%	0.27	4.04	-
F7M3_SF20_	56%	24%	0%	20%	11.6%	0.27	4.42	-

^a^ K_2_O% is the ratio of the mass of K_2_O in the alkali activator to the mass of the precursors.

## Data Availability

Data are contained within the article.
